# Multidirectional Overground Robotic Training Leads to Improvements in Balance in Older Adults

**DOI:** 10.3390/robotics10030101

**Published:** 2021-08-06

**Authors:** Lara A. Thompson, Mehdi Badache, Joao Augusto Renno Brusamolin, Marzieh Savadkoohi, Jelani Guise, Gabriel Velluto de Paiva, Pius Suh, Pablo Sanchez Guerrero, Devdas Shetty

**Affiliations:** 1 Biomedical Engineering Program, Department of Mechanical Engineering, School of Engineering and Applied Sciences, University of the District of Columbia, 4200 Connecticut Ave. NW, Washington, DC 20008, USA; 2 Department of Mechanical Engineering, School of Engineering and Applied Sciences, University of the District of Columbia, 4200 Connecticut Ave. NW, Washington, DC 20008, USA; 3 Department of Electrical and Computer Engineering, School of Engineering and Applied Sciences, University of the District of Columbia, 4200 Connecticut Ave. NW, Washington, DC 20008, USA; 4 School of Engineering and Applied Sciences, University of the District of Columbia, 4200 Connecticut Ave. NW, Washington, DC 20008, USA

**Keywords:** elderly, aging, rehabilitation robots, assistive robotics, sensory training, falls, balance

## Abstract

For the rapidly growing aging demographic worldwide, robotic training methods could be impactful towards improving balance critical for everyday life. Here, we investigated the hypothesis that non-bodyweight supportive (nBWS) overground robotic balance training would lead to improvements in balance performance and balance confidence in older adults. Sixteen healthy older participants (69.7 ± 6.7 years old) were trained while donning a harness from a distinctive NaviGAITor robotic system. A control group of 11 healthy participants (68.7 ± 5.0 years old) underwent the same training but without the robotic system. Training included 6 weeks of standing and walking tasks while modifying: (1) sensory information (i.e., with and without vision (eyes-open/closed), with more and fewer support surface cues (hard or foam surfaces)) and (2) base-of-support (wide, tandem and single-leg standing exercises). Prior to and post-training, balance ability and balance confidence were assessed via the balance error scoring system (BESS) and the Activities specific Balance Confidence (ABC) scale, respectively. Encouragingly, results showed that balance ability improved (i.e., BESS errors significantly decreased), particularly in the nBWS group, across nearly all test conditions. This result serves as an indication that robotic training has an impact on improving balance for healthy aging individuals.

## 1. Introduction

With increases in average life expectancy, and drastic projected increases in the number of older individuals, the importance of improving and maintaining balance has significant societal relevance for the aging population [1]. Falls are a major concern for all adults over 65 years old [2–4]. Additionally, aside from the act of falling, one may possess an over-concern about the anticipation of falling (i.e., lack of balance confidence and a fear of falls); this can ultimately limit one’s confidence and willingness to go about their daily activities [5,6]. Thus, how to improve balance (e.g., types of training, innovative methodologies, and devices) towards enhancing mobility and reducing falls is critically important.

For aging patient populations that had recently suffered for example a fall or a stroke, robotic training was seen as an attractive tool in that task-specific gait training could begin during the early days of rehabilitation. However, all aging populations (not solely limited to the patient population) can have imbalance leading to falls. Therefore, balance investigations need to address this issue broadly. Further, to date, robotic training has focused mainly on the impacts of gait, as opposed to impacts and implications for standing balance. Standing (static) balance is critical for everyday life and daily activities, but yet it is understudied in regard to robotic training’s effects on it.

Here, we define a robotic system as an electromechanical device with a sensor-based control loop that interacts with the human body. Previous robotics have interfaced with the human body via: the support surface (e.g., platform perturbations, treadmills or movable footplates, e.g., [7–10]), a connector on the body (e.g., pelvis or harness, e.g., [11–17]), or through distributed points (via an exoskeleton, e.g., [18–22]). Such robotic devices could be wearable (i.e., worn on the body or carried by the participant), mobile (e.g., overhead suspension, gantry or with wheels), or fixed (i.e., stationary within its environment). Further, robotic systems can be overground (i.e., the individual standing on a ‘regular’ floor surface), require the use of a treadmill, or utilize plates (e.g., standing or walking on a movable platform). Here, we will overview briefly only a subset of robotic systems. In particular, we will describe robotic systems that utilize treadmills, have a connector, and are either mobile or fixed most similar to the NaviGAITor system (to be described in “Materials and Methods”) used in our studies. Table 1 provides an overview of a subset of robotic systems with partial bodyweight support (BWS) used for gait rehabilitation. Robotic bodyweight support (or BWS) provides an unweighing of one’s body weight and can be adjusted to the individual based on their need of support, for example, while undergoing balance and rehabilitative training. The systems in Table 1 are used for robotic therapy for neurological movement disorders (e.g., stroke, spinal cord injury, cerebral palsy, and traumatic brain injury).

Early studies suggested that the use of BWS led to a better recovery of ambulation, with effects on increases in overground walking speed, endurance, and decreased physical assistance in order to walk [23–28]. Previous studies have investigated gait training using partial BWS versus non-bodyweight support (nBWS) for treadmill walking; nBWS training involves the participant bearing their full weight while donning the robotic. A study conducted by Visintin et al. [23] showed that, for patients with stroke, retraining gait while a percentage of their body weight was supported resulted in better walking abilities than for gait training with nBWS training at post 6-week training and at 3 months after the training concluded.

It had been hypothesized that task-specific locomotor training via robotic-assistance on a treadmill would result in improvements in walking. Gait orthoses, for example the Lokomat [29–31], had been utilized in numerous rehabilitation settings to treat patients with locomotor deficiencies [32]. The Lokomat robotic has an overhead harness system and attaches to the patient’s lower extremities; the Lokomat pushes the individual’s leg when it deviates as they are walking on the treadmill [33,34]. Although there was a projection that robot-assisted gait training might even replace conventional physiotherapy [35], the superiority of task-specific locomotor training has not yet been proven at an adequate level. For a long time, task-specific locomotor training appeared superior over strength training in patients who had suffered a stroke [24,36], but more recent studies concluded that a home-exercise program targeting flexibility, strength, coordination or balance, was equivalent [37]. Graham et al. [38] stated that robotic treadmill training (either BWS or nBWS), typically involves high step count, faster walking speed, and higher heart-rate intensity than overground walking training (without robotics).

Thus, treadmill training may offer increased opportunities to improve walking and balance skills. In their study, they compared walking and balance outcomes of chronic stroke survivors performing training with BWS and training with BWS including challenging mobility skills. Yet, neither BWS treadmill training nor robot-assisted gait training have fulfilled the high expectations that were initially thought by researchers and clinicians [39]. Although there was a projection for robotic training to have a large impact in advancing gait rehabilitation, there has been no indication that these methods have superior outcomes to overground training [40,41]. For example, results of the 10 min walk test pointed out that robot-assisted gait training was not superior compared to strength training. Further, there were no statistical differences between the changes in scores due to robot-assisted gait training and strength training for balance measures. However, this aforementioned study had for the first time investigated the effect robotic training on static balance; based on their assessment tools, neither intervention had an influence on static balance.

There has been general enthusiasm towards use of overground robotic rehabilitation for survivors of stroke. More recently, it had been determined that robotic treadmill gait training both with and without BWS (i.e., BWS and nBWS, respectively) led to improvements observed in, for example, walking speed and distance. Additionally, further effects were still observable post- use of the robotic treadmill training intervention [42,43]. A systematic review by Mehrholz et al. [44] showed that, compared to overground walking training, treadmill gait training (both BWS and nBWS) in ambulatory patients led to significantly improved walking speed and endurance. Additionally, the benefits persisted after the training concluded.

The studies discussed above for robotic-assisted training, have the following limitations: (1) they did not measure standing balance but instead focused almost exclusively on gait and (2) they focused solely on patient populations. However, these studies are important to discuss in that they demonstrate the current state of related research tied to robotic bodyweight supportive systems, task-specific robotic training, and overground robotic rehabilitation.

It is well-known that sensory inputs to the visual, somatosensory, and vestibular systems integrate to provide information on one’s orientation in space, balance and equilibrium. Additionally, base-of-support (BOS), the projected area between the points of contact with the ground, can greatly impact one’s stability. Thus, robotic training should investigate the incorporation of various levels of training difficulty (i.e., modifying both sensory input and BOS), as well as having both static and dynamic tasks; taken together, this could lead to improvements in one’s balance.

There have been limited studies on sensory-type training in combination with robotic training for balance. A more recent study [45] was targeted towards determining effects of sensory inputs (specifically vision) in combination with either robotic BWS or nBWS for gait training in subacute stroke survivors. In particular, it was assessed if treadmill gait training, in conjunction with visual biofeedback and BWS, would produce improved gait in subacute stroke patients compared to the same training but without visual biofeedback. It had been observed that treadmill training with acoustic biofeedback led to improved gait coordination in patients with chronic stroke [46,47]. Additionally, moderate treadmill training (i.e., 15–30 min per day, for two weeks) with visual biofeedback, was compared to exercise on a treadmill alone, in chronic stroke patients and yielded better results in improving gait cycle length, duration of gait phases, and swing phase speed [35]. However, for the early stages after stroke, this particular study did not support their initial hypothesis that visual biofeedback accompanying BWS treadmill training enabled greater improvement in walking abilities.

Previous robotic training studies did not utilize assessment tools which modified sensory information nor BOS (e.g., without vision, hard versus compliant support surface, narrowed versus wide stance), so it was unknown the effects it had in this regard. The effects the training had on balance different levels of sensory difficulty were not evaluated, however, these metrics are important in that older individuals could be prone to a fall at night, getting up at of bed in a dark room, while standing on a compliant carpeted floor, due to the limited information they are receiving visually and through their foot (somatosensory) cues. Another key limitation was that the previous studies above were solely focused on the effects of robotic training and/or partial BWS on gait. Thus, the parameters measured were solely focused on walking speed (e.g., via walk tests). Further, they did not examine standing balance nor did they assess balance confidence. Even though maintaining standing balance is important towards fall prevention, there have been very few studies on the effects of robotic training on static balance. Our overground robotic device (NaviGAITor) allowed for multidirectional movements, as opposed to simply forward walking or treadmill walking in previous studies.

Here, we explored the use of sensory training and standing and dynamic tasks, while older participants donned a multidirectional robotic system (called “NaviGAITor”). We hypothesized that for aging individuals: (1) NaviGAITor nBWS robotic training would lead to improvements in standing balance, and (2) NaviGAITor nBWS robotic training would lead to improvements in balance confidence.

## 2. Materials and Methods

All study activities were conducted within the Center for Biomechanical & Rehabilitation Engineering (CBRE) at the University of the District of Columbia. The research study protocol was approved by the Institutional Review Board (979744–1), and all participants gave their informed consent prior to participating in the study. Participants were mainly recruited via flyer postings around the University campus but also by word of mouth. Further, the University of the District of Columbia’s Institute of Gerontology disseminated flyers and information to prospective participants.

### 2.1. Participants

Thirty healthy participants (60–85 years old) enrolled in this study. Included participants needed to have a score greater than 25 on the Mini-Mental State Examination (all participants scored > 28). Further inclusion criterion was the ability to ambulate freely without the use of a cane or walker. Participants were excluded from the study if they had suffered a stroke, had reported incidents of severe heart failure or other heart conditions, had cognitive disorders, for example, impairing the understanding of and ability to follow directions, had visual field disturbances (use of glasses was permitted), disorders significantly affecting the subjects’ gait and/or balance, and/or untreated deep vein thrombosis left untreated.

**Figure 1.**
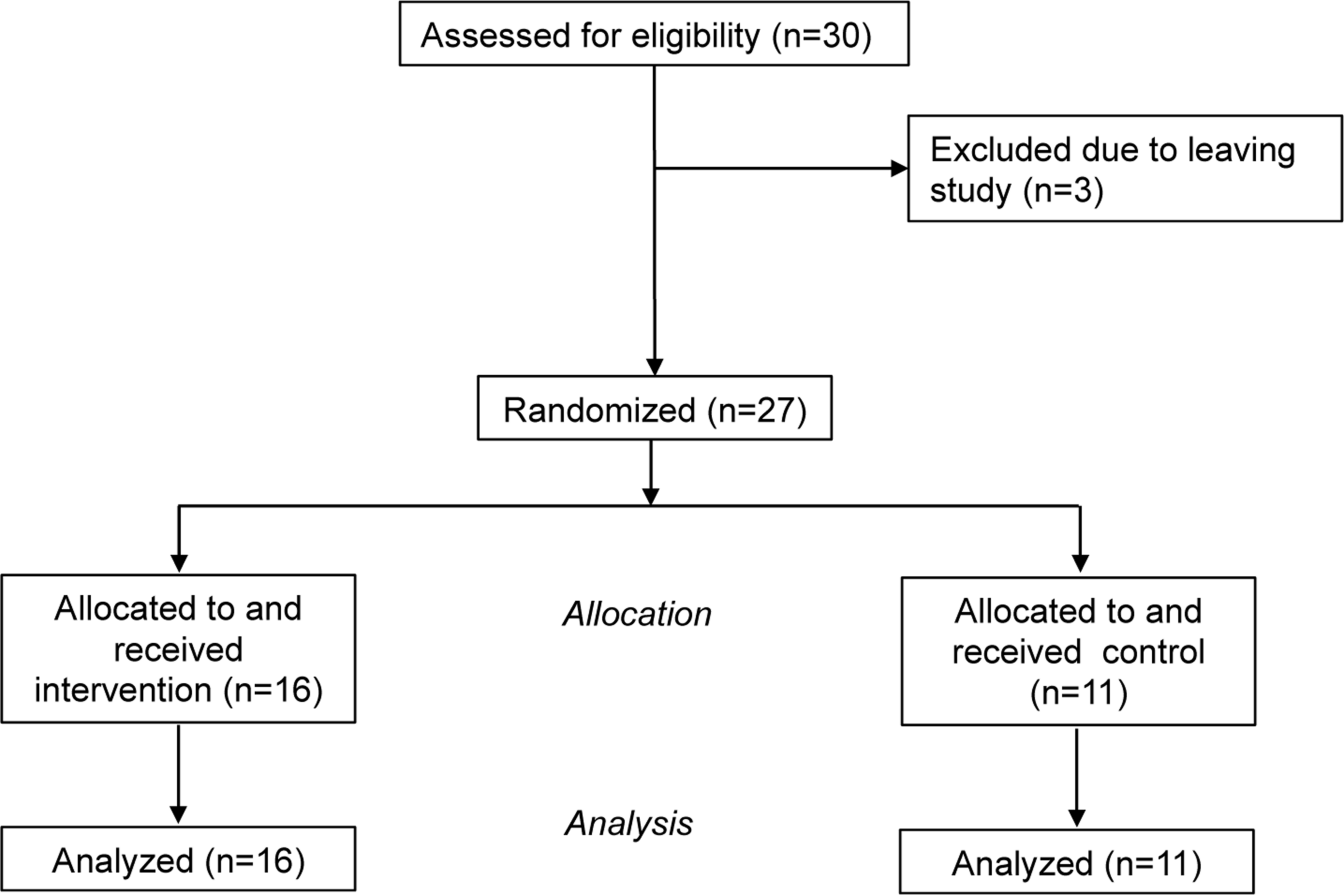
Study Flow Diagram.

The results are presented for 27 healthy: 16 nBWS (69.7 ± 6.7) and 11 controls (68.7 ± 5.0 years old). We randomized the assignment of participants to one of two parallel groups (Figure 1). Simple randomization was used, whereby the participants were allocated either to the nBWS group using the or to the control group.

### 2.2. Training Protocol

#### 2.2.1. NaviGAITor System (Used for Experimental (Intervention) Group)

**Figure 2.**
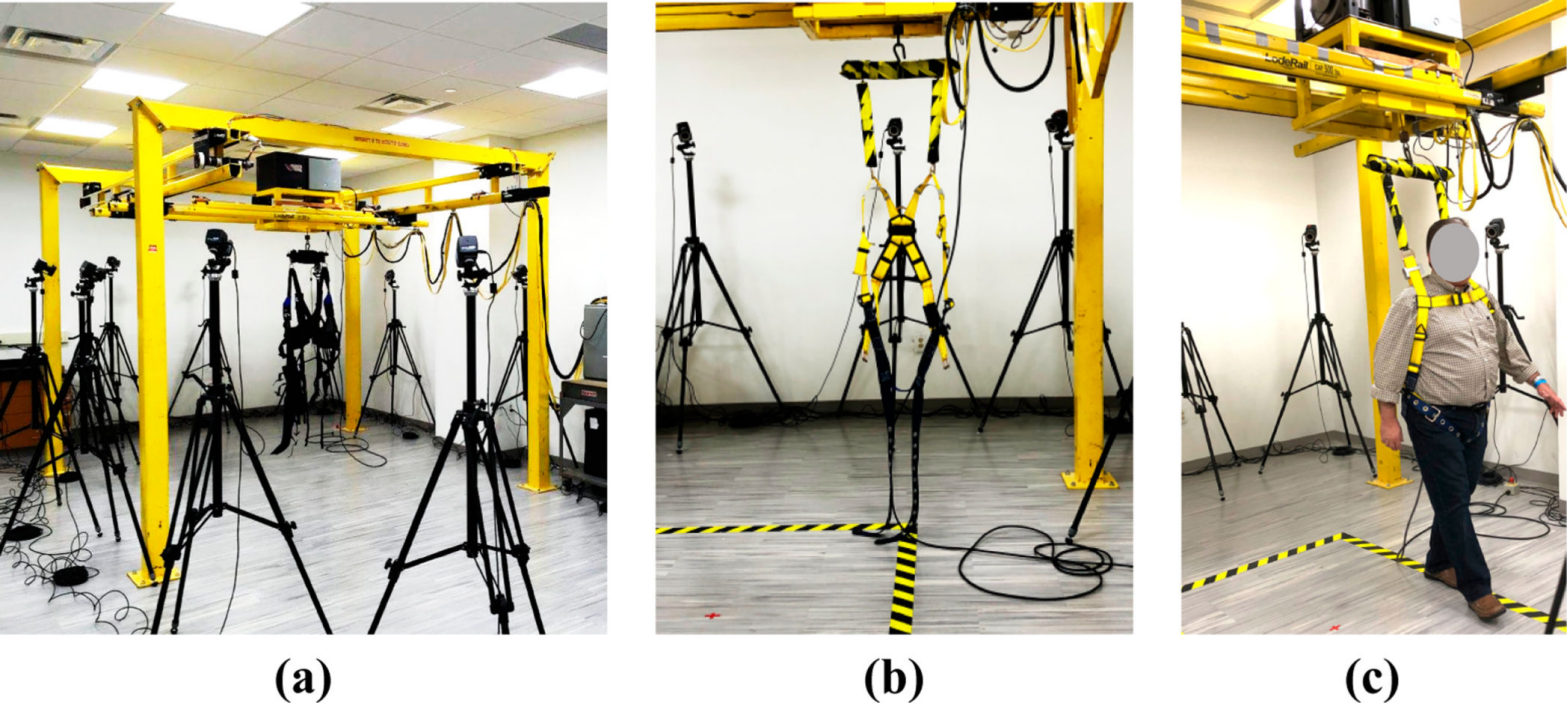
Overview of NaviGAITor robotic system: (**a**) the NaviGAITor system consisting of an I-beam support structure (frame), gantry, motor, hoist, overhead harness, as well as sensors and a control system; the vicon motion capture set up shown is not part of the NaviGAITor system; (**b**) NaviGAITor harness with torso support and two thigh straps; (**c**) demonstrative use of NaviGAITor system for overground walking and balance training.

The intervention (nBWS) group used the NaviGAITor system shown in Figure 2. The NaviGAITor is a distinctive ambulatory gait training apparatus that was designed to improve balance. This device is meant to facilitate balance training in patients, (e.g., survivors of stroke) and others who may have balance impairments. The equipment is comprised of a dynamic weight-supported gantry training system, which allows motion in the horizontal (or “XY” plane) plane and vertical axis (or “Z” axis). The device acts as an automated support structure for patients by providing full range of motion support, allowing ambulatory impaired patients and individuals to rehabilitate or improve their balance under the supervision of a physical therapist. As the individual walks, within the confines of the room-sized gantry, the hoist will directly follow the individual.

The NaviGAITor system consisted of an I-beam support structure (frame), gantry, motor, hoist, overhead harness with torso support and two thigh straps, as well as sensors and a control system. A mechatronics design approach provided the ability to transform an industrial gantry system, sensors, and actuators into a balance and gait training system. The hoist was controlled using multi-axis tilt sensors and strain gauge sensors. These sensors detected the participants’ movements (i.e., the strain gauge detected vertical movements on the hoist line and tilt sensors detected movements in the horizontal plane). The overhead gantry was motorized for movements in the horizontal planes: X (left-right/mediolateral) and Y (front-back/anterior-posterior). Closed-loop motor control was linked to feedback from the sensors. Vertical (*Z*-axis) force feedback, X and *Y* systems were integrated into a multi-axis control system.

As the person moved, the closed-loop motor control reacted to feedback from multiaxis tilt sensors on the hoist line attached to the harness. The NaviGAITor system utilized three, variable speed motors. These motors were utilized in combination with sensors to dynamically track the individual’s position and movement intent. Vertical force (rope tension) was the control variable for the *Z*-axis; this was measured via a load cell and bridge amplifier assembly. In the horizontal plane, the X and *Y*-axis movements were sensed via an accelerometer-based tilt sensor and feedback amplifier assembly. The control system was developed with features which include manual control (guided by the experimenter) and automatic control (guided by the individual’s movements), as well as an emergency mode which utilized “smart sensing” for unanticipated events (e.g., if the individual fell or lost their balance). The harness safeguarded the person from injury in the event of a fall (abrupt movement in the *Z*-axis), and gave a sense of security, while providing limited resistance to the person’s movements. In the event of a fall, the system would stop, locking the position of the three dc motors thereby supporting the individual. A control system of the type proportional and derivative was employed.

The NaviGAITor system allows the individual to move in multiple directions, different then just straight path walking as with previous robotic training systems. While the participant donned the NaviGAITor harness, the gantry system allowed them to move in in the horizontal plane. Although it was not used in this study, the overhead harness had the capacity to provide unweighing via vertical support (body weight support, BWS) of the participant while they did overground walking exercises and training. Further, the system could also move vertically.

Some features of the NaviGAITor system are summarized here:

- Rather than straight path walking which is facilitated by other existing systems (e.g., ZeroG, Lokomat, and Biodex), the NaviGAITor helps the individual to perform overground movements and walking in the horizontal plane. Motorized XY motion is used so that the individual can start, walk, and stop with minimal effort and maximum safety. While the participant donned the NaviGAITor harness, the gantry system allowed them to move within the horizontal plane.
- Although it was not used in this study, the overhead harness had the capacity to provide unweighing via vertical support (BWS) of the participant while they did overground walking exercises and training. Participants in this study did not receive BWS but had experienced nBWS secure walking attributed to the robotic device.
- Although here we only utilized movements in the horizontal plane for training, the NaviGAITor has the capacity to enable movement training in along three axes of motion without risk of falls and injuries. The system provides the ability for an individual to, for example, go up and down stairs. Further, the motorized *Z*-axis motion with self-locking allows vertical displacement under constant or variable tension and will prevent a fall. Automation prevents falls and allows emergency stops.
- The lead-lag controller algorithm and leading offset algorithm helps the individual’s movement. The individual does not have to exert additional pressure on the cable as the control algorithm guarantees a smooth movement.
- The apparatus can be configured to accommodate different therapy facilities that may require adjustments in the mechanical and computer systems to maintain uniform and consistent operation.

#### 2.2.2. Control Group

The control group involved 11 healthy participants. The control participants did not don the NaviGAITor device (described above) during their training. Instead, they were allowed to place a single index finger on the spotter’s arm during eyes closed tasks as a precautionary measure. However, they were not allowed to grab nor place significant bodyweight on the spotter’s arm.

#### 2.2.3. Training

All participants underwent a 6-week exercise routine which consisted of two, 30 min sessions/week; each participant was trained individually. Participants did not wear shoes nor footwear during both the training and the assessments described below.

Over the course of several weeks, training progressively increased in difficulty. The training protocol was the same for both control and nBWS with the exception being the use of the NaviGAITor for the nBWS group. The training involved eyes-open/closed activities for: (1) walking (tandem/wide and frontwards/backwards) as well as side-stepping left and right; (2) foam exercises (wide/tandem/isolated leg exercises); (3) walking over obstacles and a variety of foam exercises (Table 1 in [48]). During several weeks of training, participants were exposed to conditions which trained them to make use of diverse sensory information (missing or available cues) while attempting to maintain their balance during both standing and walking tasks. Visual, somatosensory, and vestibular systems affect balance. Thus, training modified vision (eyes-closed/eyes-open, respectively) as well as modified somatosensory cues (thick foam, medium density foam, hard surface) to make conditions more or less challenging, respectively. Base-of-support (BOS) was also modified between large and small (e.g., double-leg, tandem, and single-leg stances) to increase task difficulty. A larger BOS (double-leg stance) allows for greater stability whereas smaller BOS (tandem and single-leg stances) could lead to lesser stability. During the sessions, the participants worked with the principal investigator and spotters (research assistants).

### 2.3. Assessment Protocol

In order to assess participant balance, pre- and post-assessments were conducted to measure balance performance and balance confidence for each participant.

#### 2.3.1. Measures of Balance Performance

Because of the variations in support surface and BOS, we specifically chose to use the balance error scoring system (BESS) assessment, a standard assessment. The BESS utilized double-leg, single-leg, and tandem stances, on either firm or foam surfaces, as the participant stood upright with eyes-closed and hands-on hips. The BESS assessment allowed for varied levels of task difficulty (modifying support surface (or foot somatosensory cues) and BOS).

**Figure 3.**
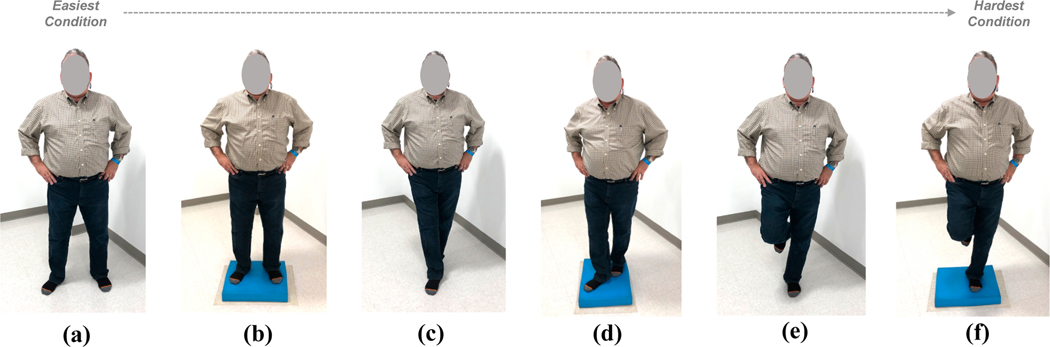
BESS Test Conditions: (**a**) double-leg stance/hard surface (DL/Hard), (**b**) double-leg stance/foam surface (DL/Foam), (**c**) tandem stance/hard surface (T/Hard), (**d**) tandem stance/foam surface (T/Foam), (**e**) single-leg/hard surface (SL/Hard), (**f**) single-leg/foam surface (SL/Foam).

To reduce learning effects, stances were presented using a latin square design. “Errors”, or deviations from upright, were counted for each of the six, 20 s trials for each condition. The BESS conditions were the following—*easiest*: double-leg stance/hard surface (DL/Hard), double-leg stance/foam surface (DL/Foam), tandem stance/hard surface (T/Hard), tandem stance/foam surface (T/Foam), single-leg/hard surface (SL/Hard), *hardest*: single-leg/foam surface (SL/Foam)). Figure 3 shows the BESS test conditions.

Some examples of BESS errors are extending hands and arms away from one’s sides (off of hips) in order to balance, opening one’s eyes in order regain their ability to balance, stepping or stumbling, crouching (hip abduction or flexion beyond 30°), remaining out of the proper testing position for over 5 s. Each error was given 1-point and errors were counted throughout each trial. A higher score (larger number of deviations) could be interpreted as lesser ability to balance, and conversely, a lower score (fewer number of deviations) could be interpreted as a better ability to balance. Ten was the maximum number of errors that were allowable for each BESS test condition. If a participant reached 10 errors, then that condition was then stopped and the value of 10 was recorded for that trial. Thus, the maximum score a participant could receive for each BESS condition was 10 (worst possible score) and minimum number of errors was 0 (best possible score).

#### 2.3.2. Measures of Balance Confidence

To assess participants’ balance confidence pre- and post-training, the ABC scale (or Activities Specific Balance Confidence scale), a standard assessment, was used. The survey questions included for example, how confident the participant was when they: walked around their house, reached for something above eye-level, bumped into people as they walked, walked on icy sidewalks and others. In terms of the scoring, 100% balance confidence would indicate a high, perceived level of balance ability, while less than 50% would indicate a low, perceived level of balance ability and 0% would indicate no balance confidence. This survey was administered both pre- and post-training, and each participant was blind to their survey responses from their previous assessment.

### 2.4. Analysis

Each participant’s data was organized, tabulated and post-processed in Microsoft Excel (Version 16.26) spreadsheets. For each group (control and nBWS), for each test condition, trials were pooled from which means and standard errors of the mean were computed. For the BESS assessment, mean BESS errors as a function of BESS stance/test condition for each group (i.e., control and nBWS) were plotted. This was done in order to determine how stance deviations (BESS errors) changed as a function of increase in stance task difficulty (BESS test conditions) for each test group, as well as for post- compared to pre-training. For the ABC scale results, we examined the mean pre- and post-training values for each group, as well as for post- and pre-training. Significant differences were observed as *p*-values < 0.05 and determined by using statistical analysis (using *t*-tests for equal sample size, unequal variance) between the pre- and post-assessments.

Further, we observed examined the percentage differences post-training compared to pre- by Equation (1). We removed the absolute value, which is typically found in the numerator, so we could observe percentage increases or decreases post- compared to pre-training. Percentage differences were computed for both the BESS and ABC data sets. Percentage decreases in BESS errors could indicate an improvement in balance while percentage increases in ABC scale results could indicate an improvement in balance confidence.


(1) $$\%difference=\frac{\left(post\;value-pre\;value\right)}{(post\;value-pre\;value)/2}*100\%$$

## 3. Results

### 3.1. Measures of Balance Performance Results

**Figure 4.**
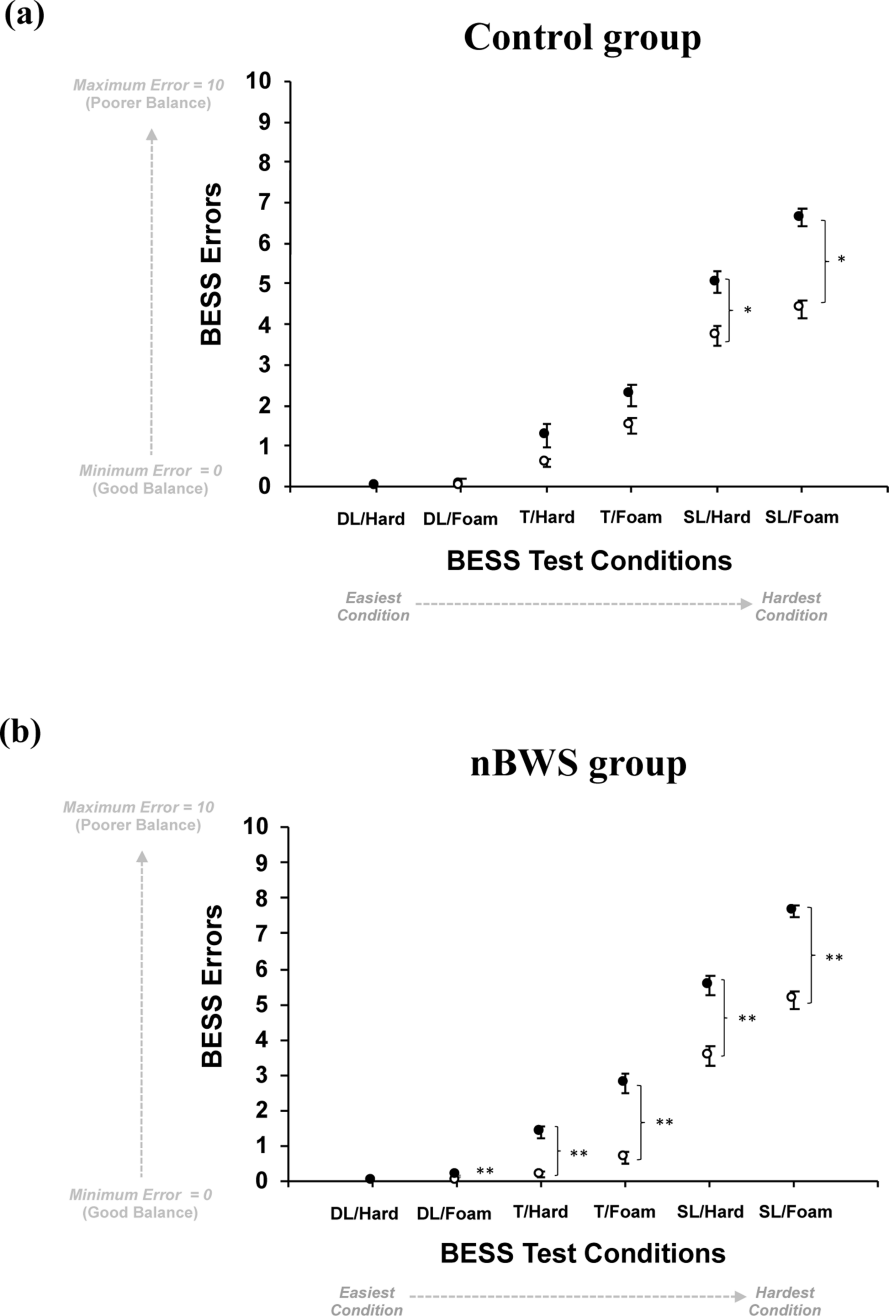
BESS errors as a function of test condition for the (**a**) control group and (**b**) nBWS (NaviGAITor) group for pre- training (filled circles) and post-training (open circles). Per condition, maximum possible errors (or stance deviations) = 10, minimum possible errors (or stance deviations) = 0. BESS test conditions are: double-leg stance/hard surface (DL/Hard), double-leg stance/foam surface (DL/Foam), tandem stance/hard surface (T/Hard), tandem stance/foam surface (T/Foam), single-leg/hard surface (SL/Hard), most challenging: single-leg/foam surface (SL/Foam). For group for each condition, means and standard error of the mean bars are shown. Significance levels are as follows: * = *p* ~ 0.02 and ** = *p* < 0.00001.

The nBWS intervention showed improvements in static balance performance as observed as decreases in BESS assessment errors, post training compared to pre. Figure 4 shows the BESS errors as a function of BESS condition (i.e., *easiest*: double-leg stance/hard surface (DL/Hard), double-leg stance/foam surface (DL/Foam), tandem stance/hard surface (T/Hard), tandem stance/foam surface (T/Foam), single-leg/hard surface (SL/Hard), *hardest*: single-leg/foam surface (SL/Foam)) for the control group (Figure 3a) and the NaviGAITor (or nBWS) group (Figure 3b). Significance levels are shown in the figure as well as within Table 2. The control group only had significant decreases in BESS errors (improvements in balance) for the two most challenging conditions (SL/Hard and SL/Foam). However, the nBWS results showed significant decreases in BESS errors (improvements in balance) for all of the test conditions except for the easiest condition (DL/Hard). Results are also shown in terms of a percent differences, calculated on the mean values, post- versus pre-training in control and nBWS groups (Table 3).

### 3.2. Measures of Balance Confidence—ABC Assessment Results

**Figure 5.**
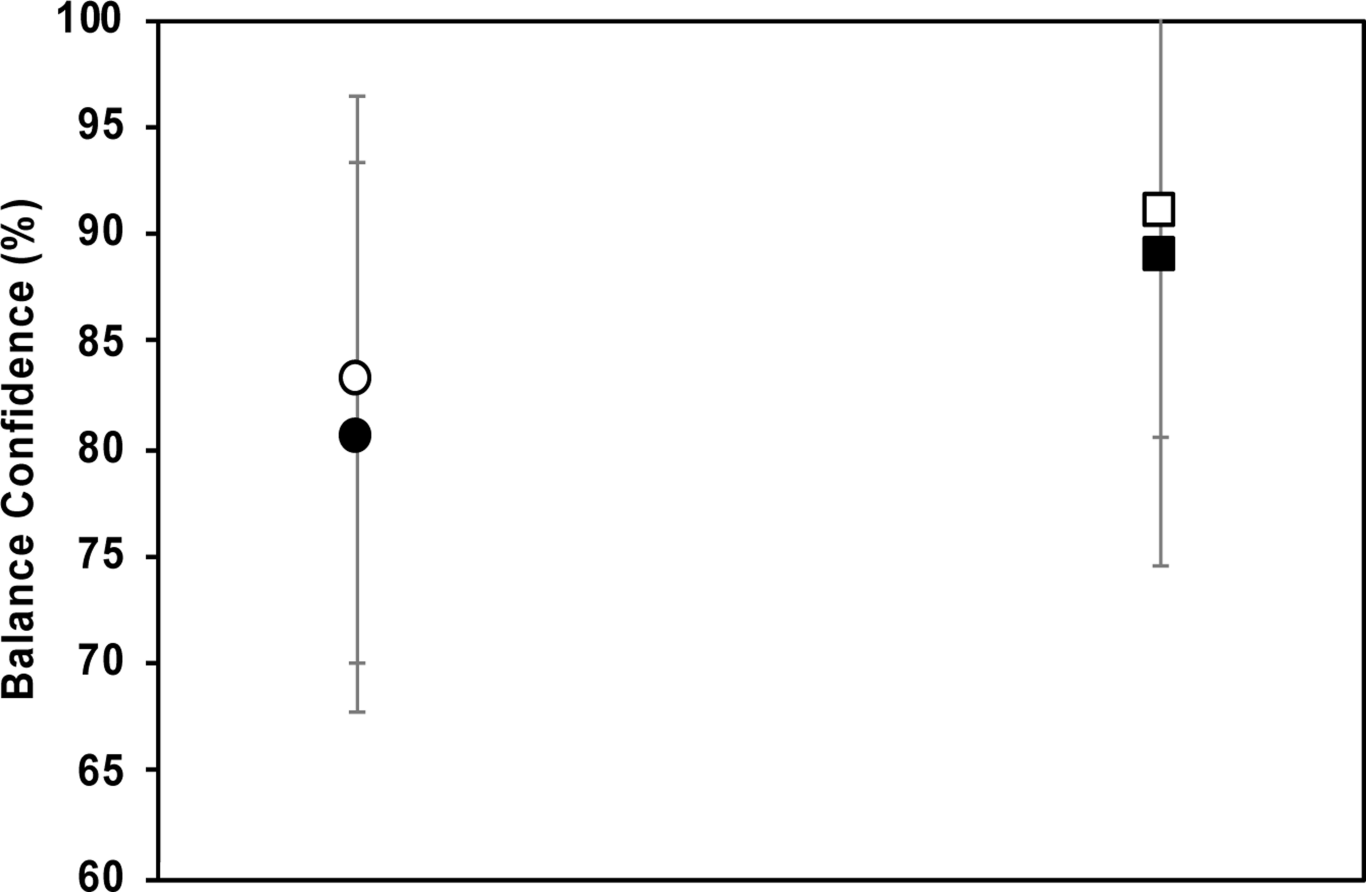
ABC balance confidence results for nBWS group (square) and control group (circle) with standard deviation bars shown for pre training (filled icon) and post training (open icon).

Measures of perceived balance ability balance confidence) were assessed through the ABC survey. Figure 5 shows that there were insignificant increases (post- versus pre-training) for both groups’ balance confidence, as well as insignificant differences between groups. In terms of percentage differences calculated on the mean values, there were a small percentage increases in balance confidence (ABC scale results) by 3.3% and 2.1% for the control and nBWS groups, respectively, post- compared to pre-training.

## 4. Discussion

The sensory-type training both with and without the NaviGAITor used here led to improvements in balance, however, improvements were much more pronounced in the nBWS group that underwent the training with the NaviGAITor system. The aging participants in the nBWS group showed improvements in balance post training, as observed by the decreases in BESS errors for nearly all conditions. Yet, the control group showed significant decreases only for the most difficult stance conditions (single leg stance on hard and foam surfaces). The improvements seen in the control group showed that some balance changes could be due to the sensory training itself, but gains were more significant with the use of a nBWS robotic system. This result may serve as an indication that robotic training is important for healthy aging individuals’ balance maintenance and improvements via task-specific repetitive training, based on the principle of neuroplasticity.

We attempted to address the existing gap on robotic training for varied sensory information during static balance in aging individuals. Our results show that sensory training could augment robotic-type training methodologies. Further, as previously stated, previous works had focused almost exclusively on patient populations. The aging population is growing rapidly and our study is an indication that this population could benefit from use of balance robotics. From our results, it is promising that non-patient populations that are potentially prone to falls (older adults) can have improved balance after robotic training. Previous studies in stroke patients had demonstrated the additional value of robot-assisted gait training combined with conventional training [49]. Further, for other patient populations (traumatic brain injury patients and those with multiple sclerosis), robot-assisted gait training may have a beneficial effect on the rehabilitation process [49]. However, for the aging population that are not patients, robotic balance training combined with sensory training may be a method towards improving their balance and should be further explored. Further, this study investigating healthy older adults forms the basis for patient population balance studies using the NaviGAITor.

Here, we did not focus on patient populations as it had been a topic of several previous works. Further, we thought it important to characterize the NaviGAITor first on healthy aging individuals. Thus, only healthy aging subjects were focused on and reported here. Other measurements and assessments in future works will be used to further probe the effects of the robotic-type training. Our results could be further substantiated with testing a greater number of participants, and thus, this result could be regarded as a baseline finding for other studies. Further, we did not follow-up several months after the intervention to see if the improved balance continued or “washed out”. It is interesting that although balance improved, balance confidence remained the same (post- versus pre-training) for each group. This could possibly mean that either longer duration training could lead to improved balance confidence or that an additional intervention, aside from the balance training, could be needed to enhance the older participant’s confidence in their abilities to do certain tasks.

Aside from patient populations, the possibility that aging individuals could utilize robotics for improving or maintaining their balance is encouraging. Our results show that nBWS support, combined with sensory training while the participant (overground) mobilized in the horizontal plane led to improvements in standing balance. This result serves as an indication that robotic training has an impact on improving balance for healthy aging individuals. The effectiveness of nBWS training via the NaviGAITor could be determined in a long-term clinical study, however, the results presented here are encouraging towards potential robotic-type methodologies to improve balance in older adults.

## Figures and Tables

**Table 1.** Overview of robotic systems used for gait and balance.

Robotic Device	Image	Description
Biodex Unweighing System ^1^	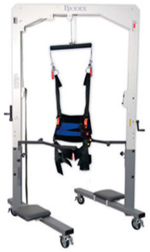	The Biodex Unweighing system allows for partial bodyweight support (BWS) of the patient. Additionally, overground walking and movements which replicate natural ambulation are possible while using this device. This device does not allow for vertical movements.
Lokomat System ^2^	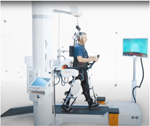	The Lokomat is a robotic treadmill training device that allows for partial BWS of the patient. There is a harness and robotic (automated gait orthosis device) that attaches to the person while they walk on a treadmill. The concept behind use of this device is that, by continuous repetition of movements, it can train patients to re-learn normal walking.
ZeroG System ^3^	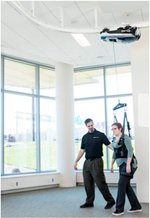	The ZeroG is a partial BWS system that moves along a driven trolley attached to an overhead rail system. The system allows the patient to do overground walking and vertical movements. The rail system is typically attached to ceilings over 9 feet high but can be designed without ceiling integration.
KineAssist Walking and balance system ^4^	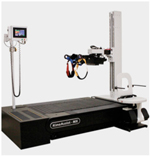	This KineAssist robotic device consists of a hip brace and harness that connects to an actuation system. It provides partial BWS and postural torques on the torso while following patient’s walking motions overground in forward, rotation and sidestepping directions.
Autoambulator ^5^	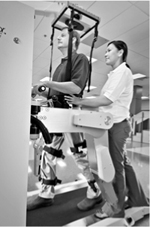	The Autoamblator system incorporates robotic assistance, with connectors at the lower limbs, to simulate normal walking motion. A harness provides partial BWS to the patient while they walk over a treadmill. This device does not allow for vertical movements.
NaviGAITor	(See Figure 2)	The NaviGAITor is an ambulatory suspension and rehabilitation apparatus system designed by D. Shetty and experimented by the authors. It is a newer device for research and clinical applications. It enables exercise and movement training in all three planes of motion. These features are made possible because of mechatronic design methodology.

1 Picture: https://www.dotmed.com/listing/physical-therapy-unit/biodex/unweighing-system,-offset/896111.

2 Picture: http://www.hocoma.com/us/solutions/lokomat/.

3 Picture: https://www.aretechllc.com/.

4 Picture: https://www.woodway.com/products/kineassist/.

5 Picture: https://healthscopemag.com/health-scope/autoambulator/. (accessed on 6 July 2021).

**Table 2.** Significant decreases (post training compared to pre) for each BESS test condition within both control and nBWS groups; blank cells indicate that changes were insignificant for those BESS test conditions.

Group	DL/Hard	DL/Foam	T/Hard	T/Foam	SL/Hard	SL/Foam
Control					df = 16, t = −2.46, *p* = 0.026	df = 12, t = −2.72, *p* = 0.019
nBWS		df = 15, t = −11.54, *p* < 0.00001	df = 29, t = −7.32, *p* < 0.00001	df = 29, t = −7.32, *p* < 0.00001	df = 16, t = −2.46, *p* < 0.00001	df = 27, t = −8.23, *p* < 0.00001

**Table 3.** Percent differences for each BESS condition in control and nBWS groups post- compared to pre-training.

BESS Condition	Control	nBWS

DL/H	Undefined	undefined
DL/F	200%	−200%
T/H	−69.1%	−153.8%
T/F	−41.1%	−122.9%
SL/H	−30.1%	−43.7%
SL/F	−40.8%	−39.2%
